# Multiple Large Placental Lakes in an Accreta Placenta Previa: A Rare Case Report

**DOI:** 10.1155/carm/5079470

**Published:** 2026-01-30

**Authors:** Yahia Ranjous, Fadi Alhalak, Ali Deeb, Abdullah Ismail, Wessam Taifour, Dema Adwan

**Affiliations:** ^1^ Faculty of Medicine, Damascus University, Damascus, Syria, damascusuniversity.edu.sy; ^2^ Gynecology and Obstetrics Hospital, Damascus University, Damascus, Syria, damascusuniversity.edu.sy

**Keywords:** case report, hysterectomy, multiple large placental lakes, placenta accreta, placenta previa

## Abstract

**Introduction:**

Placental lakes are smooth and blood‐filled cavities of varying sizes observed during routine ultrasound examination of pregnant women.

**Presentation of Case:**

This report describes a case of a 35‐year‐old Syrian woman who complained of vaginal bleeding at 39 weeks of gestation. The patient had a history of seven cesarean sections. Echography revealed anterior accreta placenta previa and 4 large placental lakes measuring 10, 8, 4, and 4 cm. Complete hysterectomy was performed, and the patient was discharged without complications.

**Discussion:**

The importance of this case lies in the presence of four placental lakes, with the largest reaching a size of 10 cm, accompanied by an accreta placenta previa, which is rare.

**Conclusion:**

Maternal and fetal complications remain controversial, as some studies have suggested direct effects on the health of the mother and fetus, whereas others have refuted any correlation.

## 1. Introduction

The global incidence of placenta accreta has increased, primarily due to the growing prevalence of uterine scarring following cesarean sections. Although less common, placenta accreta may also develop in uteri without prior scarring [1]. Modern advances in imaging technologies, including echography and magnetic resonance imaging (MRI), have facilitated the detection of various placental abnormalities, such as placenta previa, and the spectrum of placenta accreta spectrum (PAS), which can worsen maternal and fetal outcomes [2, 3]. Placental lakes are hypoechoic, irregularly shaped, blood‐filled cavities of varying sizes in the placenta [4]. The exact effect of these placental lakes on fetal and maternal well‐being remains controversial, as research findings vary. Some studies have reported no significant outcomes, whereas others have indicated potential consequences such as fetal growth restriction [5–15].

In this case, we report the case of a 35‐year‐old pregnant woman with an accreta placenta previa containing four large placental lakes measuring 10, 8, 4, and 4 cm. We followed the SCARE checklist in writing this case report [16].

## 2. Case Presentation

A 35‐year‐old Syrian woman, gravida 8, para 7, presented to the emergency department at 39 weeks of gestation based on the date of her last menstrual period. The patient complained of vaginal spotting. She had undergone seven previous cesarean sections, the most recent of which was one and a half years ago for the delivery of a twin pregnancy. In addition, she reported two episodes of bleeding in the seventh and eighth months of pregnancy, but did not seek medical attention. Otherwise, there were no previous miscarriages or dilatation and curettage procedures, and she denied any significant medical conditions, medication usage, or allergies. The patient did not adhere to her regular follow‐up and, therefore, did not undergo an anatomical ultrasound during the second trimester. The vital signs were stable (blood pressure: 100/68 mmHg, pulse rate: 88 beats per minute, oxygen saturation: 95%, and temperature: 37.1°C). Abdominal examination revealed that the height of the uterine fundus was consistent with the gestational age without any uterine contractions. Fetal heart rate was detected using Doppler echography and recorded at 139 beats per minute. Abdominal echography revealed a single viable fetus in a breech presentation with a biparietal diameter consistent with 38 weeks of gestation and femur length consistent with 37 weeks of gestation. The amniotic fluid volume was within the normal range (AFI: 11 cm). However, partial coverage of the cervical os by the anterior placenta previa was observed. The myometrial thickness behind the placenta was less than 1 mm, with irregular uterine margins exerting pressure on the bladder, resulting in a bulge and loss of the retroplacental clear space, suggesting a PAS. Several wide, irregular hypoechoic placental lakes were observed, with the first measuring 10 cm, the second measuring 8 cm, and the third and fourth measuring 4 cm (Figure 1). Speculum examination revealed a closed cervix, with blood dripping from the internal os. Subsequently, the patient was admitted to the labor ward with continuous monitoring of vital signs and fetal heart activity using cardiotocography (CTG). Two intravenous lines were opened, blood samples were collected for laboratory investigations, and a urinary catheter was inserted to monitor the urinary output. The laboratory results revealed anemia, indicated by low hemoglobin (8.2 g/dL) and hematocrit (24.8%) levels, platelet count of 205 × 10^3^/μL, creatinine of 0.84 mg/dL, urea of 25 mg/dL, INR of 1, and partial thromboplastin time (PTT) of 25.6 s.

Figure 1Echography imaging of the placenta revealing the placental lakes.

**Figure:**
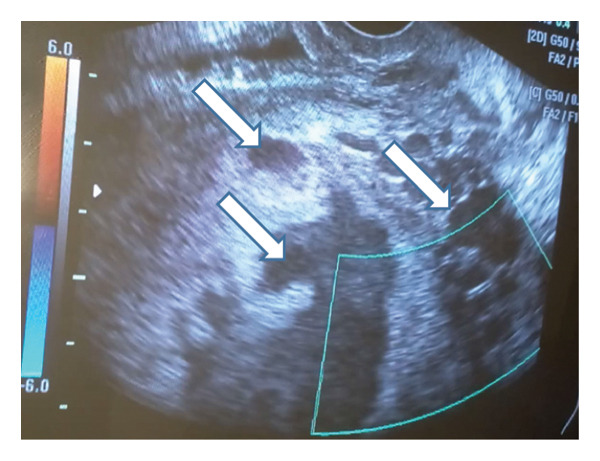
(a)

**Figure:**
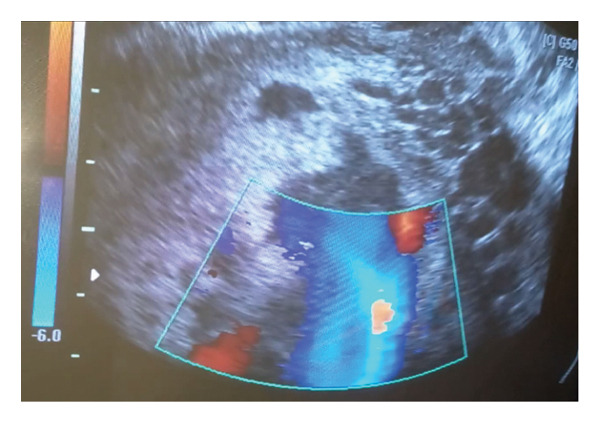
(b)

**Figure 2:**
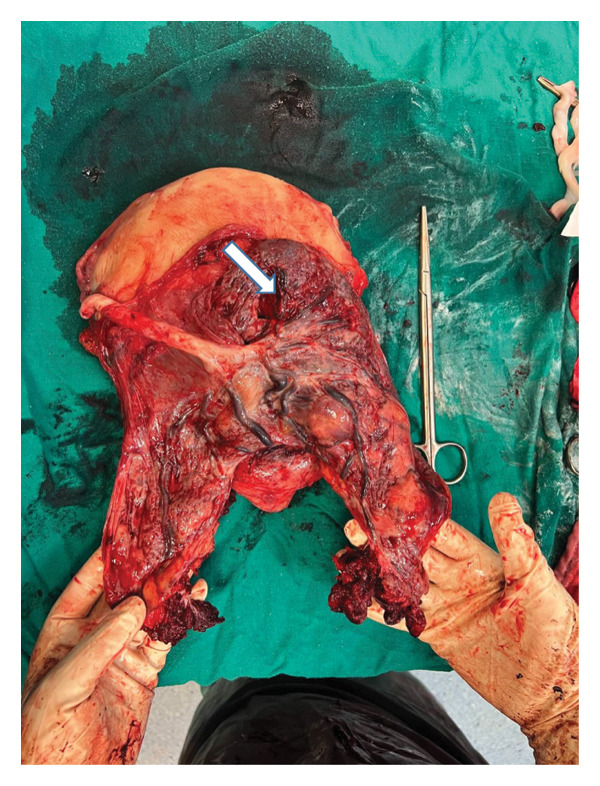
Macroscopic view of the resected uterus.

The decision to perform surgery was made after explaining the case to the patient and gaining her consent, and the placenta was found to reach the serosa layer of the uterus. The FIGO stage was determined based on echography and surgical findings, which indicated that the tumor invaded the entire uterine wall, reaching and breaching the serosa without invading the bladder or other nearby organs. Therefore, the PAS grade according to FIGO was classified as Grade 3a. An incision was made in the upper part of the uterus to prevent harm to the placenta, and a healthy baby weighing 3200 g was delivered and handed over to the pediatrician. The infant’s Apgar score was 8 at one minute and 10 at five minutes after birth. The decision was made to perform a complete hysterectomy with adnexal preservation. The uterus was extracted from the abdomen and the umbilical cord was clamped and left inside the uterus, keeping the placenta in its location. The incision on the upper part of the uterus was sutured before the hysterectomy was completed (Figure 2). During surgery, four units of blood and three units of fresh frozen plasma were transfused. The patient was transferred to the intensive care unit for close monitoring of vital signs and urinary output. The postoperative recovery was uneventful, and the patient was transferred to the ward on the second day. She was discharged from the hospital on the third day, along with her child, in good and stable condition.

## 3. Discussion

Placental lakes are vascular spaces filled with maternal blood that appear hypoechoic on ultrasound and typically result from perivillous fibrin deposition with cystic changes within areas of subchorionic fibrin [15].

The literature includes several reports of isolated large placental lakes [5, 6, 17, 18], including those reported by Has. R. et al. with dimensions of 10 × 10 × 10 cm, and another case of a large placental lake recorded by Di Donato et al. measuring 10 × 6.5 × 5 cm. What sets this case apart is the presence of four placental lakes measuring 10, 8, 4 and 4 cm, concomitant with the presence of an accreta placenta previa.

While surgical hysterectomy is the standard management for PAS, modern conservative approaches have emerged in recent years to preserve the uterus such as the modified one‐step conservative uterine surgery (MOSCUS) [19].

The impact of large placental lakes measuring more than 5 cm on fetal health remains a topic of debate, with some studies indicating no significant pathological effects [7, 8, 14]. However, other studies have indicated that large placental lakes result in fetal growth restriction, sudden fetal death, and fetal cardiac disturbances [5, 6, 9–12]. Hwang et al. and Cooley et al. noted that placental lakes were accompanied by vaginal bleeding and threatened miscarriages [12, 13]. Surgical management for PAS disorders is frequently linked to adverse neonatal outcomes, such as reduced gestational age, lower birthweight, and lower Apgar score at 5 min. These complications are particularly pronounced in cases requiring emergency surgical management [20]. Our patient had not experienced bleeding until the third trimester, and the baby was healthy without complications despite the presence of four placental lakes.

The principal limitation in this case is the lack of a histopathological examination following surgery, which was not performed due to economic constraints.

## 4. Conclusion

Our study highlights the simultaneous association of four different‐sized placental lakes with an accreta and previa placenta. The potential side effects on the fetus remain controversial and warrant further investigation.

**Nomenclature**
PAS: Placenta accreta spectrum
MRI: Magnetic resonance imaging

## Author Contributions

All authors participated in the writing of the case.

## Funding

The authors did not receive funding for this paper.

## Disclosure

All authors revised and approved the final manuscript.

## Ethics Statement

The Ethical Committee of the Faculty of Medicine at Damascus University does not require ethics approval for case reports.

## Consent

Written informed consent was obtained from the patient for publication of this case report and accompanying images. A copy of the written consent is available for review by the Editor‐in‐Chief of this journal on request.

## Conflicts of Interest

The authors declare no conflicts of interest.

## Data Availability

The data that support the findings of this study are available from the corresponding author upon reasonable request.
